# Multibeam Characteristics of a Negative Refractive Index Shaped Lens

**DOI:** 10.3390/s20195703

**Published:** 2020-10-07

**Authors:** Salbiah Ab Hamid, Nurul Huda Abd Rahman, Yoshihide Yamada, Phan Van Hung, Dinh Nguyen Quoc

**Affiliations:** 1 Faculty of Electrical Engineering, Universiti Teknologi MARA, Shah Alam 40450, Selangor, Malaysia; 2 Malaysia-Japan International Institute of Technology, Universiti Teknologi Malaysia, Kuala Lumpur 54100, Malaysia; yoshihide@utm.my; 3 Faculty of Radio Electronic Engineering, Le Quy Don Technical University, Hanoi City 100000, Vietnam; phanvanhung@tcu.edu.vn (P.V.H.); dinhnq@mta.edu.vn (D.N.Q.)

**Keywords:** lens antenna, negative refractive index, multibeam, beam scanning

## Abstract

Narrow beam width, higher gain and multibeam characteristics are demanded in 5G technology. Array antennas that are utilized in the existing mobile base stations have many drawbacks when operating at upper 5G frequency bands. For example, due to the high frequency operation, the antenna elements become smaller and thus, in order to provide higher gain, more antenna elements and arrays are required, which will cause the feeding network design to be more complex. The lens antenna is one of the potential candidates to replace the current structure in mobile base station. Therefore, a negative refractive index shaped lens is proposed to provide high gain and narrow beamwidth using energy conservation and Abbe’s sine principle. The aim of this study is to investigate the multibeam characteristics of a negative refractive index shaped lens in mobile base station applications. In this paper, the feed positions for the multibeam are selected on the circle from the center of the lens and the accuracy of the feed position is validated through Electromagnetic (EM) simulation. Based on the analysis performed in this study, a negative refractive index shaped lens with a smaller radius and slender lens than the conventional lens is designed, with the additional capability of performing wide-angle beam scanning.

## 1. Introduction

In recent years, there has been a rapid development of antennas to meet the advanced mobile technology requirements. In the 5G mobile technology system, millimeter wave is used, which produces a smaller cell size and requires a multibeam radiation pattern in order to achieve massive Multiple Input Multiple Output (MIMO) operation [1,2] with the purpose to serve massive number of users, consistent interconnectivity and larger capacity. At millimeter wave, the base station size becomes less than 30cm. So, aperture antennas such as reflector antennas and dielectric lens antennas become promising candidates as compared to the present array antennas. For multibeam operation, the dielectric lens antenna can achieve very good performance by designing the lens using Abbe’s sine condition [3], which is validated further in this paper.

There were several studies conducted to investigate the multibeam application using the lens antenna. The Luneburg lens antenna is one of the commonly used designs. However, major issues of the conventional Luneburg lens are large and heavy. In [4], a Luneburg lens with an operating frequency of 1–8 GHz was proposed. In the paper, multilayer Luneburg lenses were fabricated with the diameters of 0.6 and 1.0 m and weighing of 5 and 21 kg, respectively. A number of feed antennas was placed around the lens to produce multibeam with the same beam shapes. Beam scanning was achieved by switching the feed around the surface of the lens. The traditional Luneburg lenses weight has been reduced up to 8–10 times; however, in 5G base station application, this weight and size are not practical. A flat Luneburg lens antenna with a geometry of 80 × 5 mm^2^ was designed to operate at Ka-band [5]. A linear antenna array with 11 E-shaped patch antenna elements is employed to feed the lens antenna. The lens is formed by rectangular shaped concentric six layered dielectric materials with a permittivity ranging from 1.74 to 10 to achieve higher aperture efficiency. This lens antenna produces a realized gain of 14.2 dBi. In total, 11 beams are produced for ±60° beam scanning. However, a lens antenna employing natural dielectric material is known to produce a thicker lens size, and thus is not suitable for base station application. In order to reduce the lens thickness, a metamaterial lens concept was proposed [6]. Lens structure became concave and reduced the lens thickness. As for metamaterial lens fabrication, a lens of refractive index of *n* = −1 was shown in [7]. The structure is obtained by using 100 unit cells of dielectric resonator structure, 0.5 of f/D with lens aperture of 5.7 λ (156 mm) at an 11 GHz operating frequency. The achieved multibeam radiation pattern is shown in [8]. To achieve a good multibeam characteristic, Abbe’s sine condition design is proposed in this paper, and comparison with the energy conservation technique is analyzed.

**Figure 1:**
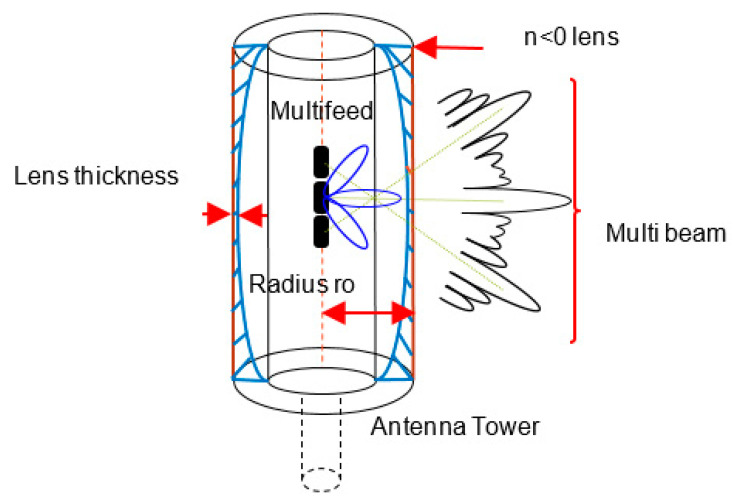
Proposed mobile base station structure.

In this paper, the application of Abbe’s sine design and energy conservation design are employed to develop a metamaterial lens. Based on the lens shaping equation, the possibility of the negative refractive index design is ensured. In order to solve the design equations, a MATLAB program is developed. The design accuracies of the MATLAB program are estimated using ray tracing results and calculation of the refraction angles on the lens surfaces. For validation, the multibeam radiation pattern is calculated using a commercial electromagnetic simulator, High Frequency Structure Simulator (HFSS). Figure 1 shows the proposed base station structure.

## 2. Design Method

In designing the lens surface, the ray tracing method was employed. All rays passing through the aperture plane are designed to be parallel to the z-axis in order to achieve a flat wave front at the aperture plane.

### 2.1. Lens Configuration

**Figure 2:**
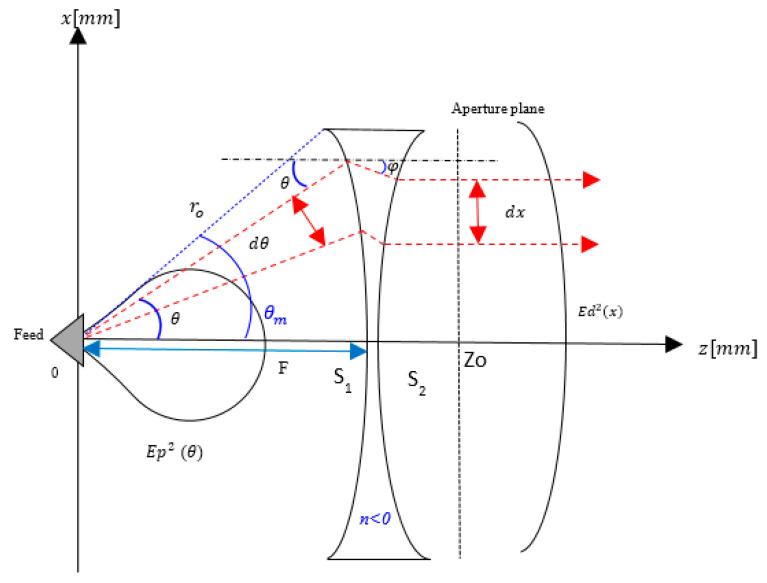
Lens configuration and parameters.

Figure 2 shows the proposed antenna configuration and the associated radiation parameters. The feed radiator radiates signal towards the negative refractive index shaped lens, and the result is observed at the aperture. The detailed description of the parameters is shown in Table 1.

### 2.2. Lens Shape Equations

#### 2.2.1. Aperture Distribution Designing

Based on Figure 2, at surface 1, S_1_ of the lens, Snell’s law is given by Equation (1) [9].

(1) $$\frac{dr}{d\theta }=\frac{rnsin\left(\theta -\varphi \right)}{ncos\left(\theta -\varphi \right)-1}$$

At the lens surface 2, the expression for the slope $\frac{dz}{dx}$ can be derived from the condition that all exit rays after refraction are parallel to the z- axis, as shown in Equation (2). The $\frac{dz}{dx}$ expression can be separated into $\frac{dz}{d\theta }$ and $\frac{dx}{d\theta }$, as shown in Equation (2) for variable change from *dx* to dθ.

(2) $$\frac{dz}{dx}=\frac{nsin\left(\varphi \right)}{1-ncos\left(\varphi \right)},\;\frac{dz}{d\theta }=\frac{nsin\left(\varphi \right)}{1-ncos\left(\varphi \right)}\frac{dx}{d\theta }$$

The equal condition of ray path length can be expressed in Equation (3) where Lt indicates the total path length from the feed to the aperture plane.

(3) $$Lt=r+n\left(\frac{x-rcos\varphi }{cos\varphi }\right)+z_{o}-z=\mathrm{constant}$$

By using this equation, the variable φ in Equations (1) and (2) can be expressed by the variable θ. Then, through simplification, the variable of Equations (1) and (2) becomes only θ. The electric power conservation at the ray is composed of dx and dθ

(4) $$\frac{dx}{d\theta }=\frac{Ep^{2}\left(\theta \right)}{\int_{0}^{\theta m}Ep^{2}\left(\theta \right)d\theta }\;\frac{\int_{0}^{Xm}Ed^{2}\left(x\right)dx}{Ed^{2}\left(x\right)}$$

The three differential Equations (1), (2) and (4) determine the lens shape in the MATLAB program. For the electric field intensity at feed radiator, $Ep^{2}\left(\theta \right),$ Equation (5) is implemented. Meanwhile, Equation (6) is used for aperture illumination distribution, $Ed^{2}\left(x\right).$

(5) $$Ep^{2}\left(\theta \right)=cos^{m}\left(\theta \right)$$

(6) $$Ed^{2}(x)=[(1-\left(1-\frac{1}{C}\right){\left(\frac{x}{X_{m}}\right)}^{2}){]}^{p}$$

#### 2.2.2. Abbe’s Sine Condition

**Figure 3:**
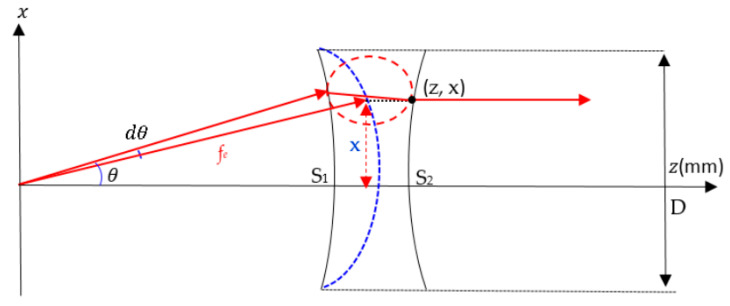
Abbe’s sine condition.

In optic, a collimating lens can be designed to be a coma free for a limited scan by imposing the Abbe’s sine condition. Coma refers to the aberration inherent to certain optical designs or due to imperfection in the lens or other components that results in off-axis point sources. This condition is automatically fulfilled if the inner surface of a conventional waveguide lens is spherical. The condition in the red circle in Figure 3 interprets the Abbe’s sine condition. When the initial and the final ray are extended, these rays are intersecting inside the lens on a circle radius of f_e_ [2]. Abbe’s sine law in Equations (7) and (9) are applied in the MATLAB program to design the shaped lens.

(7) $$x=f_{e}sin\left(\theta -d\theta \right)$$

For dθ << 1, Equation (7) becomes

(8) $$x=f_{e}sin\theta$$

(9) $$\frac{dx}{d\theta }=f_{e}cos\theta$$

### 2.3. Program Flow Chart

**Figure 4:**
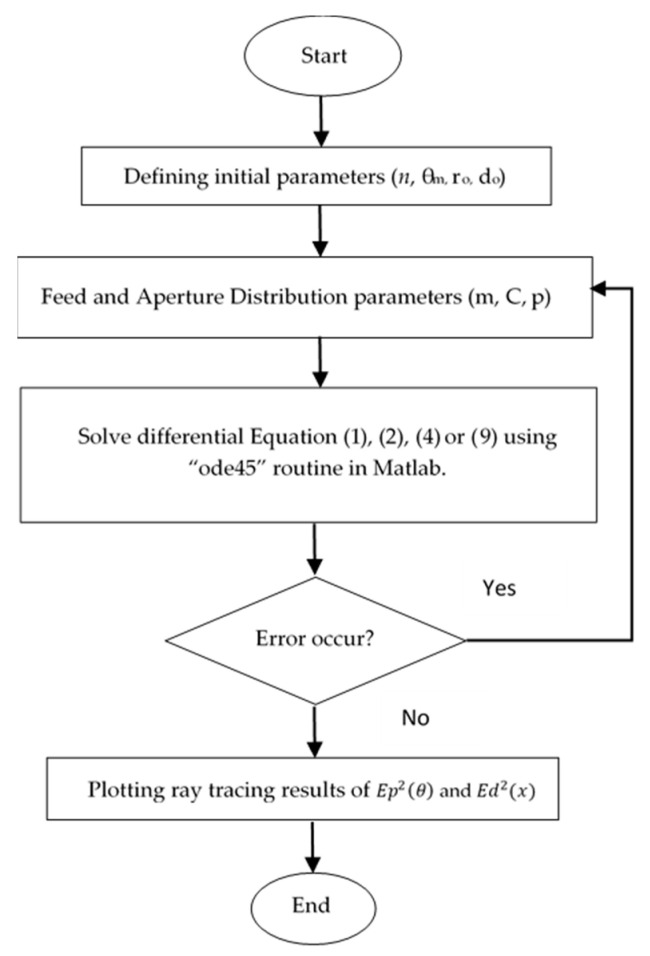
MATLAB Program.

**Figure 5:**
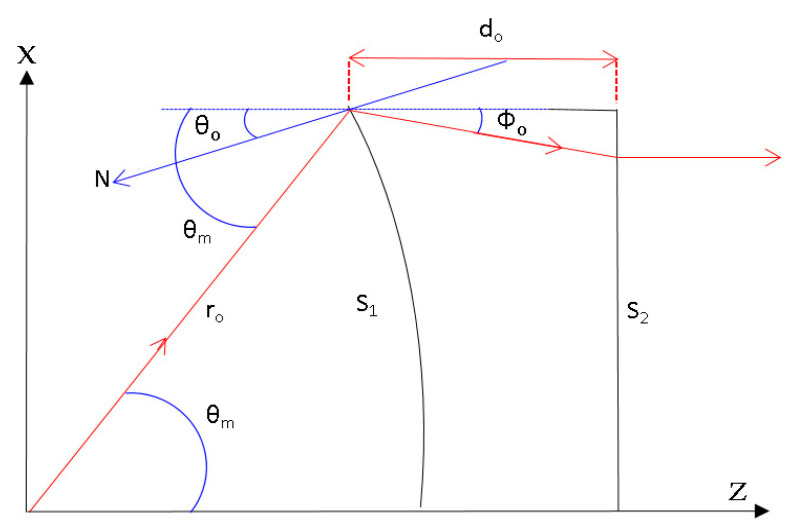
Initial parameters at the lens edge.

A flow chart in Figure 4 represents the processes involved in negative refractive index lens design using MATLAB software. Program codes were developed based on the equations and formula that were previously described in Section 2.2.1 and Section 2.2.2. The initial parameters (*n*, *θ_m_*, *r_o_*, *d_o_*) determined at the lens edge are shown in Figure 5. The equations for feed radiation pattern, $Ep^{2}\;\left(\theta \right),$ and aperture distributions, $Ed^{2}\left(x\right)$, are given. Next, differential Equations (1), (2) and (4) or (1), (2) and (9) are solved by the MATLAB routine of “ode45”.

The explanation for the initial input values is shown in Figure 5. $\theta_{0}$ is an important parameter that determines the lens thickness where $d_{0}$ is the initial thickness.

## 3. MATLAB Shape Design

### 3.1. Simulation of Energy Conservation Law

A negative refractive index lens is designed based on the energy conservation law by using Equations (3) and (4) in the MATLAB shape design program.

#### 3.1.1. Aperture Distribution Design

**Figure 6:**
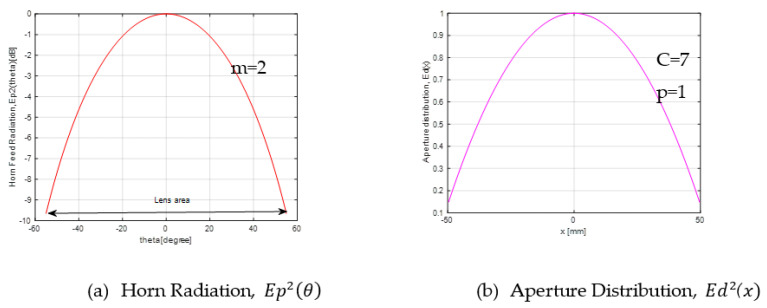
Energy conservation design.

Figure 6a shows the horn feed radiation, $Ep^{2}\left(\theta \right)$. The lens area is about −55° to 55° at −10 dB. It means that the area that is covered by the horn radiation beam is from −55° to 55°. The aperture distribution, $Ed^{2}\left(x\right)$, is shown in Figure 6b, which represents the edge level of the aperture distribution.

**Figure 7:**
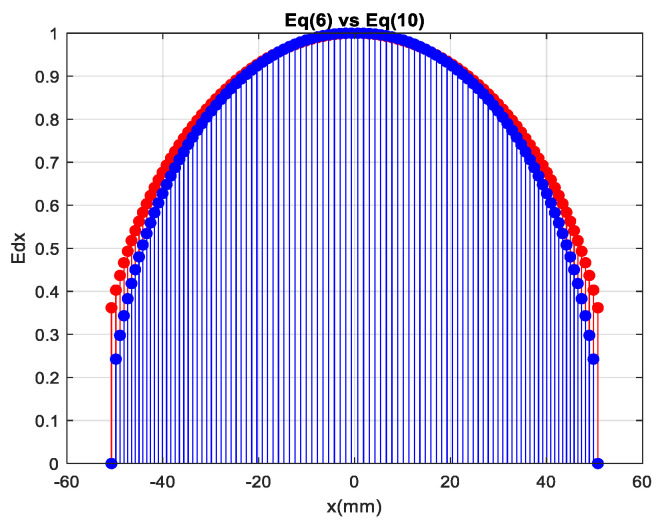
Comparison of aperture distribution.

Aperture distribution can be expressed as in (10). When Equation (10) is compared with theoretical Equation (6) in Figure 7, it is observed that both equations have a good agreement with each other.

(10) $$Ed^{2}\;\left(x\right)=\frac{d\theta \;}{dx}\;Ep^{2}\;\left(\theta \right)$$

**Figure 8:**
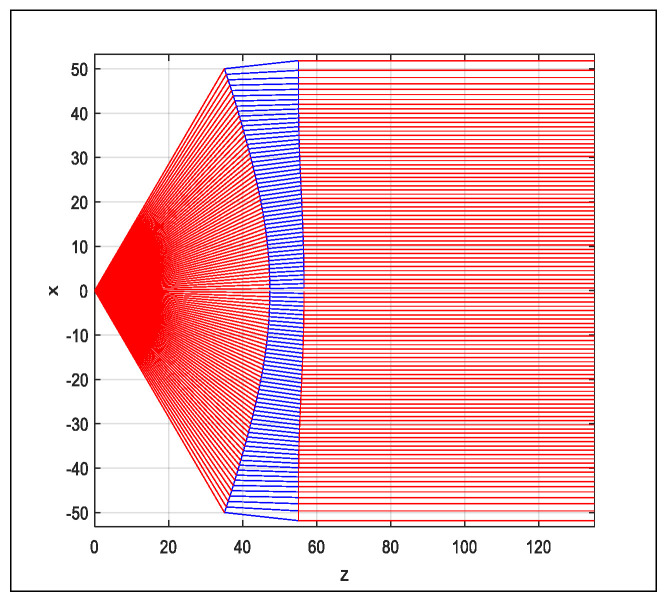
Shaped lens.

Considering all the design parameters stated previously, the shaped lens was obtained as illustrated in Figure 8. For the designed lens in Figure 8, the accuracy of the MATLAB program was validated by calculating the incident angle, $\theta_{i}$ and refracted angle, $\theta_{r}$ at each surface of the lens. The refractive index value, n, was manually determined using the Snell’s Law in (10). The calculated n value is expected to be similar to the n value (n = $-\sqrt{2}$), which is used in the MATLAB design program. The lens parameters are tabulated in Table 2.

(11) $$n_{1}sin\theta_{i}=n_{2}sin\theta_{r}$$

**Figure 9:**
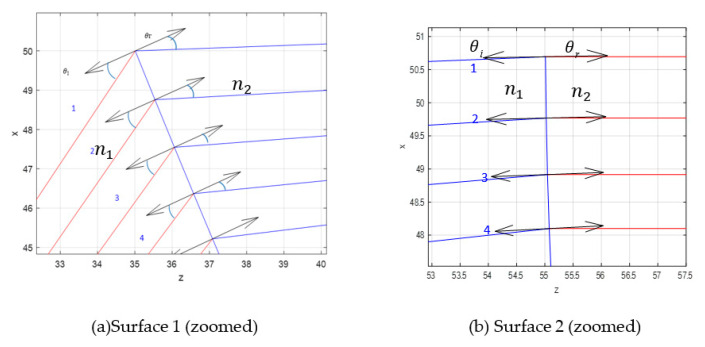
Rays in and rays out.

Table 3 represents the calculated n values considering the four first rays coming from the shaped lens in Figure 9a,b. The $n_{1}$ and $n_{2}$ values were calculated based on Equation (11) and compared to the exact n value that is used in MATLAB which is $n=-\sqrt{2}$. It is noticed that there is a small difference between the calculated values due to some errors that occurred during angle determination.

#### 3.1.2. Abbe’s Sine Condition

**Figure 10:**
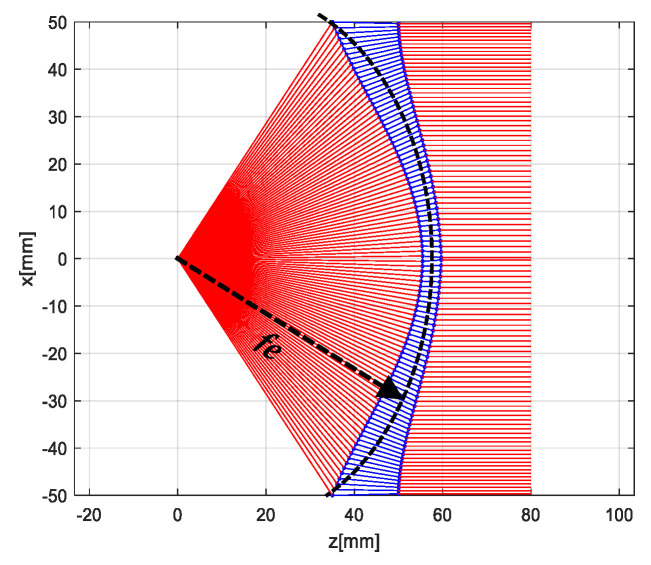
Abbe’s sine shaped lens.

The same process was repeated for Abbe’s sine Law by using Equations (7) and (9) in the MATLAB design program. The shaped lens is shown in Figure 10. The lens parameters are tabulated in Table 4.

**Figure 11:**
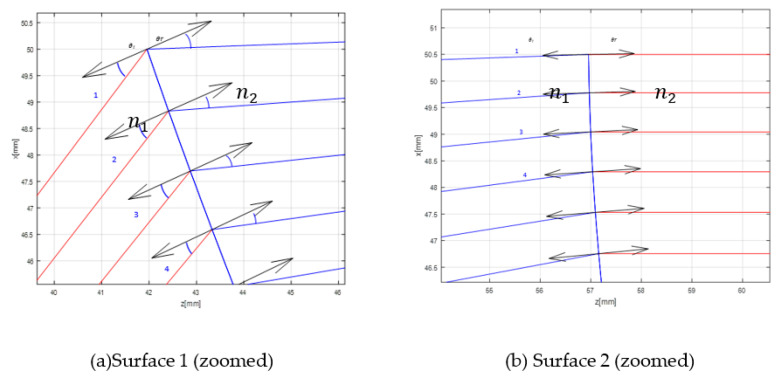
Rays in and rays out.

Table 5 represents the calculated n value considering the four first rays from the shaped lens in Figure 11a,b. A small difference between the calculated values and the exact value used in MATLAB may be due to errors during angle determination.

## 4. EM Simulations for Radiation Characteristics

### 4.1. Horn Feed

In order to calculate a negative refractive index material, the HFSS Simulator was used. In MATLAB, a point source was used to represent the feed, but in HFSS, a horn antenna was used to represent the feed radiator. Table 6 shows the simulation parameters using HFSS.

**Figure 12:**
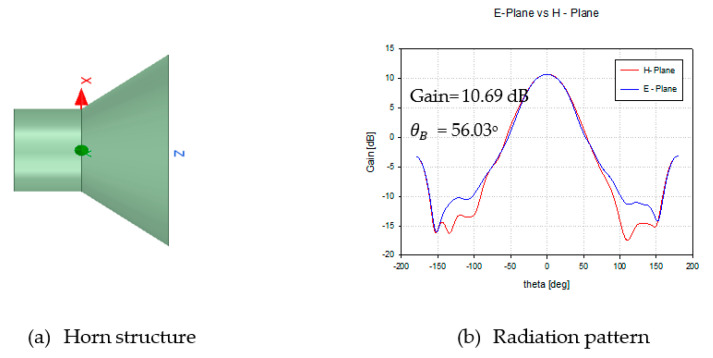
Performance of horn antenna as a feed radiator.

A conical horn antenna is designed to operate at 28 GHz as shown in Figure 12a with the radiation pattern illustrated in Figure 12b.

### 4.2. Energy Conservation Law

#### 4.2.1. On-Focus

**Figure 13:**
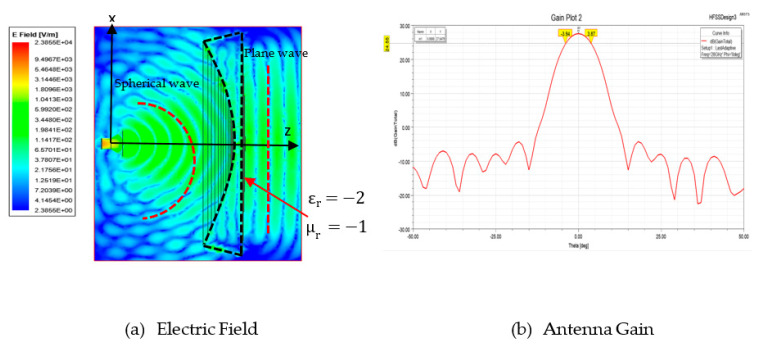
Energy conservation shaped lens performance.

The designed shaped lens structure was simulated for performance evaluation. The lens structure has a permittivity of $\varepsilon_{r}=-2$ and permeability $\mu_{r}=-1$. Figure 13a,b show the electric field distribution and the antenna gain, respectively. From Figure 13a, it is observed that the lens transformed the spherical wave into the plane wave. The obtained parameters are shown in Table 7. The theoretical value shows the uniform aperture case. This lens antenna structure produced a 27.55 dB gain with a beamwidth of 7.81°. The efficiency of the structure is about 66%.

#### 4.2.2. Off-Focus

**Figure 14:**
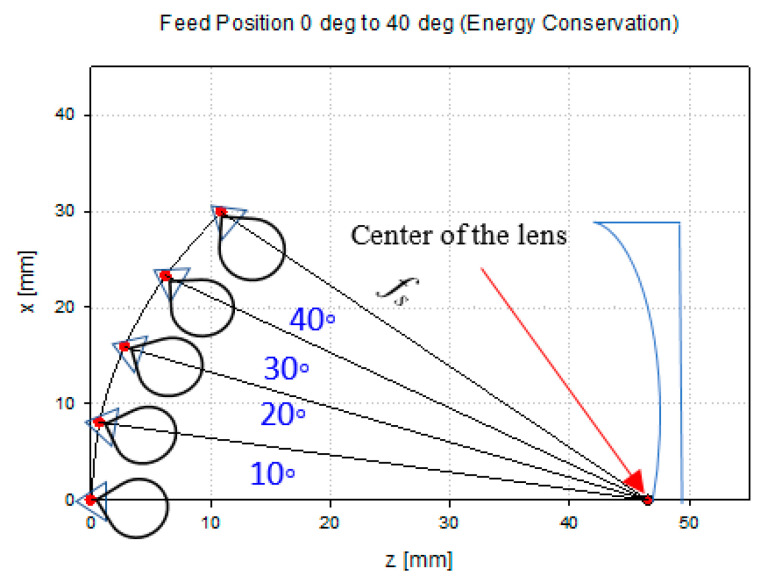
Feed position arrangement.

In order to prove that the designed lens shape is suitable for wide-angle beam scanning, multibeam characteristics are investigated in this paper. The feed horn position shifts spherically by 10° using the formula of F=fs, where F is the distance between the feed position and the center of the lens, while fs is the focal length. The off-focus position is illustrated in Figure 14. 

**Figure 15:**
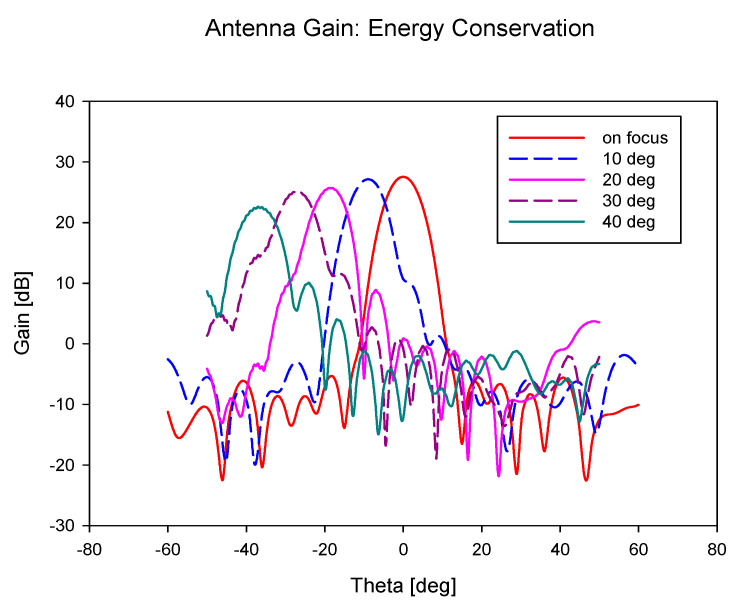
Antenna gain for scanning angle 0° to 40°.

The results of antenna gain for different feed positions is illustrated in Figure 15 and Table 8. It is observed from the graph that there is no significant gain reduction for all the scanning angles. The scanning losses are all less than 3 dB, which indicates that beamwidths are consistent for all scanning angles. It can be concluded that this structure is suitable for wide-angle beam scanning.

**Figure 16:**
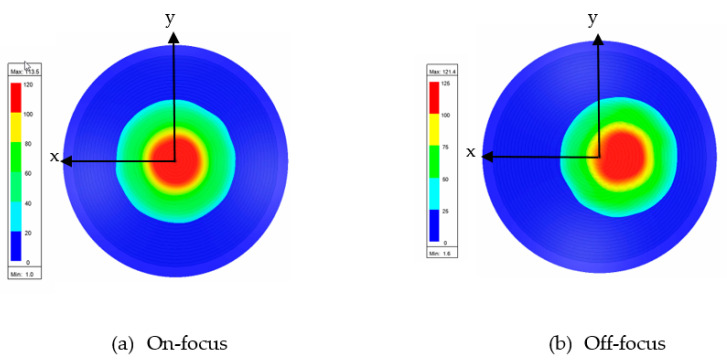
Electric intensity distribution.

**Figure 17:**
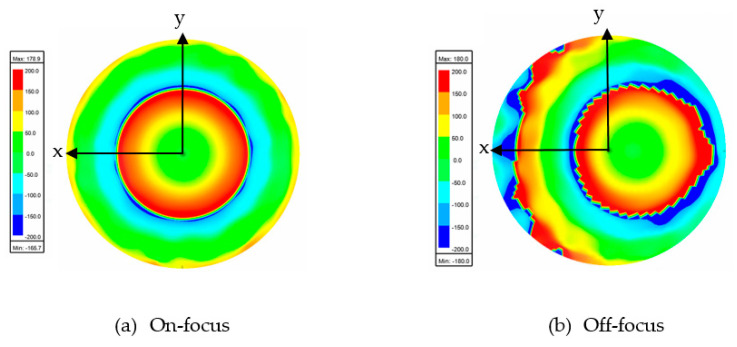
Electric phase distribution.

Figure 16 and Figure 17 show the results of the electric distribution in magnitude and phase, respectively, performed through near-field calculation. For on-focus condition, both intensity and phase distributions show uniform and symmetrical characteristics as shown in Figure 16a and Figure 17a, respectively. In off-focus condition, the uniform characteristics are expected to be disturbed by some distortions due to the non-linearity in phase; thus, the behaviors are non-symmetrical, as observed in Figure 16b and Figure 17b. Both electrical intensity and phase are slightly distorted when the feed is displaced from its original position.

### 4.3. Abbe’s Sine Law

#### 4.3.1. On-Focus

**Figure 18:**
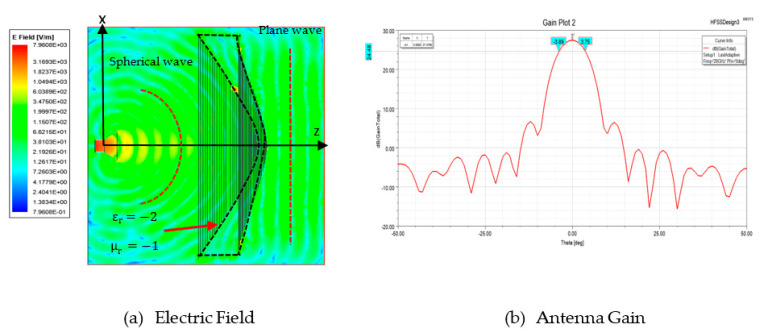
Abbe’s sine shaped lens performance.

For Abbe’s sine condition, a shaped lens structure with the permittivity value of $\varepsilon_{r}=-2$ and permeability value of $\mu_{r}=-1$ was simulated as shown in Figure 18a. The performance of the designed lens is illustrated in Figure 18b. It is observed that this Abbe’s sine shaped lens produced a gain of 27.48 dB. The beamwidth is 7.64°. This lens also has 65% efficiency, as shown in Table 9.

#### 4.3.2. Off-Focus

**Figure 19:**
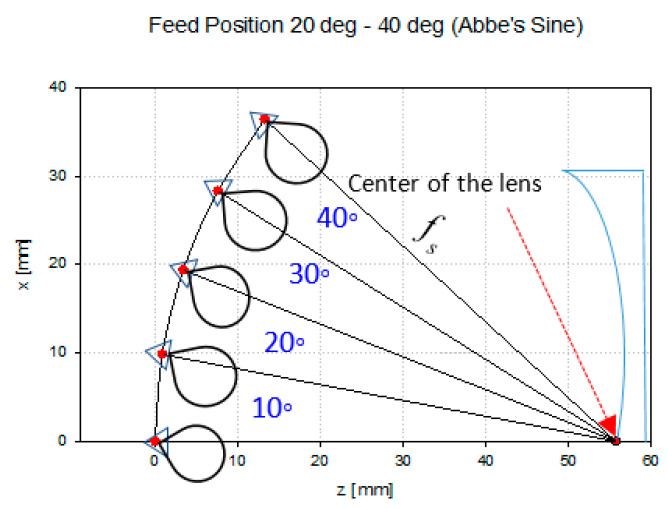
Feed position arrangement.

**Figure 20:**
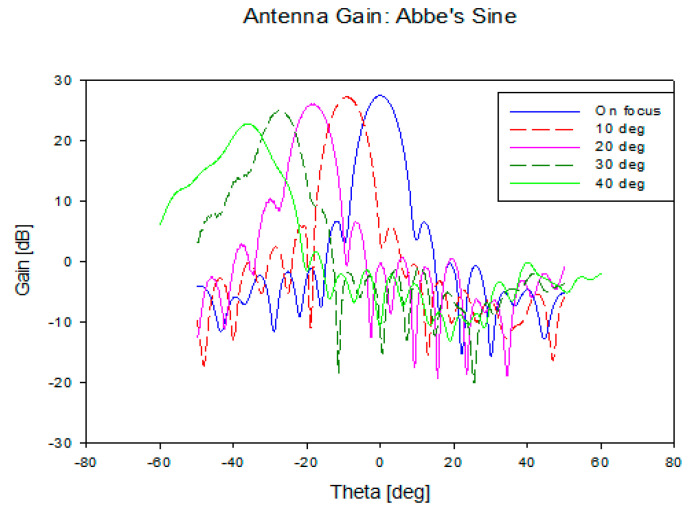
Antenna gain for scanning angle 0° to 40°.

For multibeam investigation, the feed position of the horn antenna is varied from 0° to 40° with 10° intervals as illustrated in Figure 19. The same condition in energy conservation (F=fs) was applied for Abbe’s sine shaped lens. The results for all scanning angles are represented in Figure 20 and Table 10. The gain is slightly lower as compared to the energy conservation shaped lens. However, there is no significant gain reduction for every scanning angle. The beamwidths are also consistent for all scanning angles.

**Figure 21:**
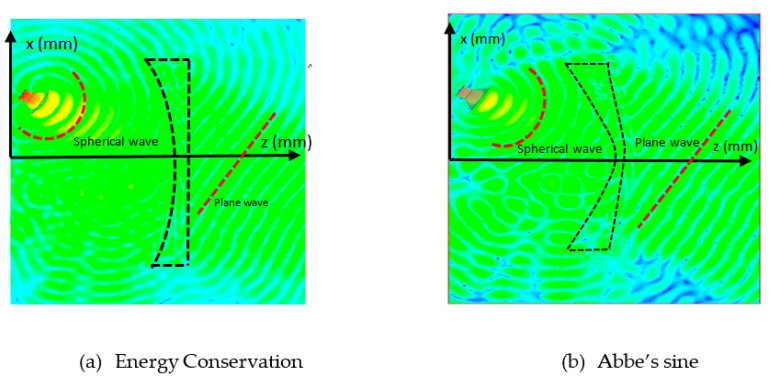
Electric field distribution during off-focus.

Figure 21a,b show the results of the electric field distribution during off-focus for Energy Conservation and Abbe’s sine lens, respectively.

**Figure 22:**
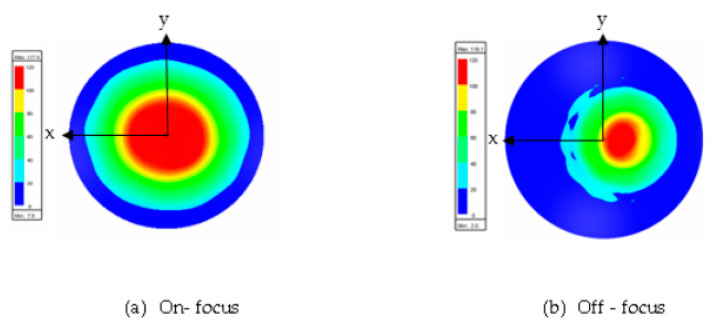
Electric intensity distribution.

**Figure 23:**
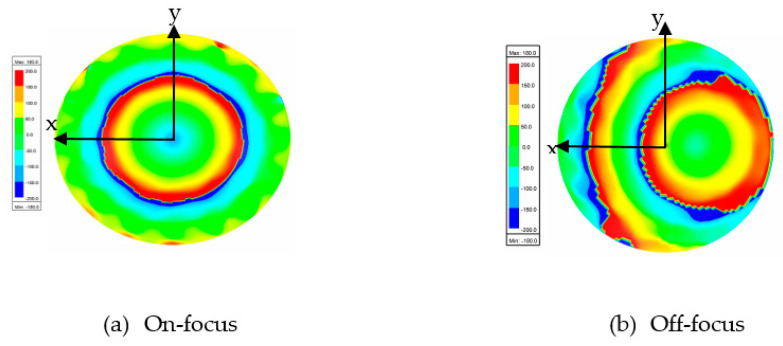
Electric phase distribution.

Figure 22 and Figure 23 show the results of the electric distribution in magnitude and phase, respectively, for Abbe’s sine lens. For on-focus condition, both intensity and phase distributions exhibited uniform characteristics as shown in Figure 22a and Figure 23a, respectively. Figure 22b and Figure 23b present off-focus condition, which shows that both electrical intensity and phase are slightly distorted. The feed displacement from its original position affects the electrical intensity and the phase distribution linearity.

**Figure 24:**
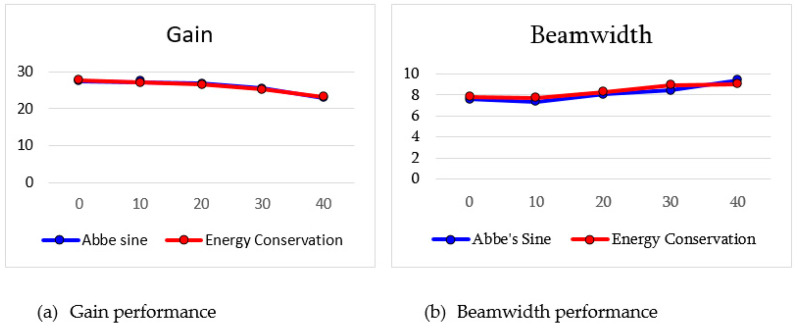
Performance comparison for both types of lens.

The performance for both types of lens are compared in Figure 24a,b. In terms of gain and beamwidth, the performances of both lenses are similar: there is not much difference, as shown in Figure 24a below.

### 4.4. Feed Position Analysis

**Figure 25:**
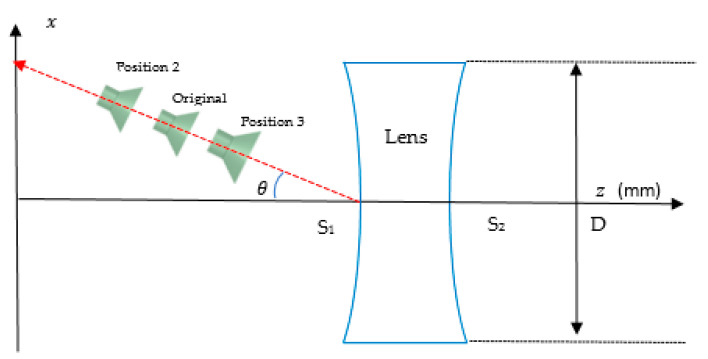
Feed position analysis arrangement.

In this section, the performance of the feed position for scanning angles of 20° until 40° is analyzed. The performance of the designed energy conservation lens and Abbe’s sine lens is evaluated when the feed is located closer and farther from the lens. The original position is F=fs as shown in Figure 25. *θ* is the scanning angle which is varied from 20° to 40°. The original position is the calculated position based on the shaped lens. Position 2 is where the feed is placed farther from the lens, while position 3 is where the feed is placed closer to the lens.

**Figure 26:**
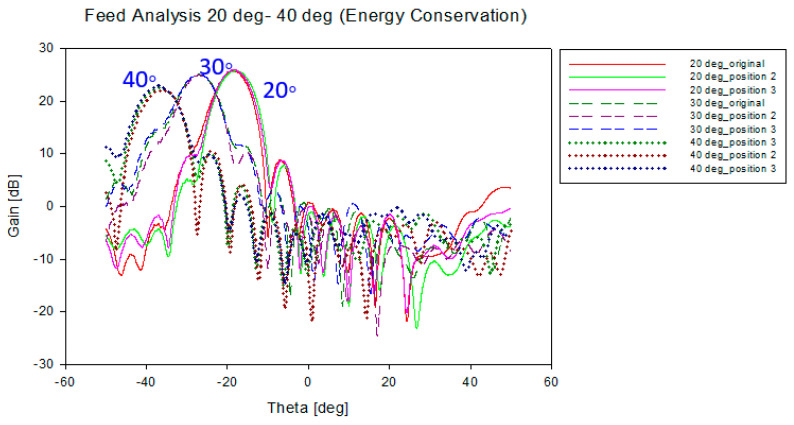
Antenna gain for all feed position for energy conservation lens.

**Figure 27:**
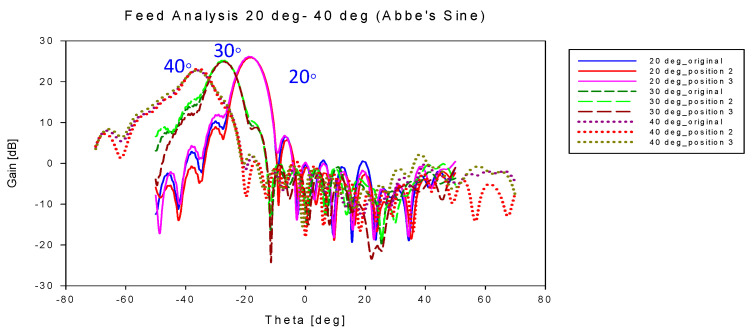
Antenna gain for all feed position for Abbe’s sine lens.

Figure 26 and Figure 27 show the performance of the designed energy conservation lens and Abbe’s sine lens antenna for all feed positions from 20° to 40° scanning angle, respectively. At each scanning angle, there is no significant difference in gain, beamwidth and shift angle between the three feed positions. It can be seen that these two types of designed lens perform consistently for all the feed positions. 

Table 11 shows the overall performance for both types of lens at 20°, 30° and 40° scanning angle. From all the results shown in Figure 26, Figure 27 and Table 11, it can be seen that all the positions along the lines satisfied the F=fs condition with optimum performance. It shows that the F=fs condition and both lens structures are suitable for multibeam applications. Hence, this structure can be applied for 5G mobile base station.

## 5. Conclusions

Two types of negative refractive indexes lens were designed in MATLAB software and simulated by using the electromagnetic simulator ANSYS HFSS. Both the energy conservation lens and Abbe’s sine lens offer high gain and narrow beam width characteristics with 66% and 65% efficiency, respectively. A 27.55 dB maximum gain is achieved for the energy conservation lens and 27.48 dB for Abbe’s sine lens. Moreover, both shaped lenses provide optimum results for beam scanning up until 40°. High gain, narrow beam width and wide-angle beam scanning capability are the key elements for 5G application. Thus, it can be concluded that these two types of refractive index lens are good candidates for 5G mobile base station application.

## Figures and Tables

**Table 1:** Antenna parameters.

Parameter	Description
n	Refractive index
$Ep^{2}\left(\theta \right)$	Feed pattern
$Ed^{2}\left(x\right)$	Aperture Distribution
$\theta_{m}$	Angle from feed to the lens edge

**Table 2:** Lens parameters.

Parameters	Value
Focal length (mm)	46.56
$\theta_{m}$ (°)	55
$\theta_{o}$ (°)	−24
n	$-\sqrt{2}$
C	7
d	20
Diameter (mm)	100

**Table 3:** Calculated *n*-value.

Ray in	θ_*i*_ (°)	θ_*r*_ (°)	$n_{2}$	Ray out	θ_*i*_ (°)	θ_*r*_ (°)	$n_{1}$
1	32	19	1.62	1	0.2	0.3	1.49
2	31	19	1.58	2	0.7	1.0	1.42
3	32	20	1.54	3	1.0	1.5	1.49
4	30	20	1.46	4	1.5	2.0	1.33

**Table 4:** Lens parameters.

Parameters	Value
*fe* (mm)	56.62
$\theta_{m}$ (°)	55
$\theta_{o}$ (°)	−24
n	$-\sqrt{2}$
C	7
d	15
Diameter (mm)	100

**Table 5:** Calculated n-value.

Ray in	θ_*i*_ (°)	θ_*r*_ (°)	$n_{2}$	Ray out	θ_*i*_ (°)	θ_*r*_ (°)	$n_{1}$
1	28	21	1.31	1	0.8	1	1.25
2	27	19	1.39	2	1.6	2	1.25
3	25	17	1.45	3	2	3	1.50
4	24	15	1.57	4	3	4	1.33

**Table 6:** Simulation parameters using High Frequency Structure Simulator (HFSS).

Parameters	Description/Value
Boundary Condition	Radiation Boundary
Refractive index,	−1.4142 ($\sqrt{-2}*\sqrt{-1}$)
Permittivity, $\varepsilon_{r}$	−2
Permeability, $\mu_{r}$	−1

**Table 7:** Lens structure simulation result.

	Theoretical	Simulation
Gain (dB)	29.35	27.55
$\theta_{B}$ (°)	8.01	7.81
ΔG (dB)	0	−1.80
***η*** (%)	100	66

**Table 8:** Simulation results of multibeam.

θ (°)	Gain (dB)	$\theta_{B}$ (°)
0	27.55	7.81
10	27.14	7.72
20	26.50	8.31
30	25.18	8.97
40	23.09	9.06

**Table 9:** Simulation results.

	Theoretical	Simulation
Gain (dB)	29.35	27.48
$\theta_{B}$ (°)	8.01	7.64
ΔG (dB)	0	−1.87
***η*** (%)	100	65

**Table 10:** Simulation results of different feed positions.

θ (°)	Gain (dB)	$\theta_{B}$ (°)
0	27.48	7.64
10	27.19	7.37
20	26.73	8.08
30	25.54	8.43
40	22.92	9.43

**Table 11:** Performance for all positions at 20°, 30° and 40° scanning angle.

*θ*		Focal Length (mm)		Gain (dB)		Beam Width (◦)		SLL (dB)		Shift Angle (◦)	
	Position	Energy	Abbe	Energy	Abbe	Energy	Abbe	Energy	Abbe	Energy	Abbe
20	Original	46.56	56.62	26.50	26.73	8.31	8.08	−17.15	−17.95	−18	−18
	Position 2	48.68	58.74	26.12	26.55	8.36	7.95	−16.63	−17.93	−17	−18
	Position 3	44.44	54.50	26.93	26.66	7.99	8.08	−16.39	−15.93	−17	−18
30	Original	46.56	56.62	25.18	25.54	8.97	8.43	−11.65	−11.37	−25	−27
	Position 2	48.68	58.74	24.92	25.25	8.95	8.50	−11.60	−11.36	−25	−27
	Position 3	44.44	54.50	25.57	25.39	9.02	8.66	−12.24	−11.53	−26	−27
40	Original	46.56	56.62	22.61	22.78	9.48	9.34	−8.05	−7.22	−37	−36
	Position 2	48.68	58.74	22.13	23.18	9.50	9.20	−5.98	−7.94	−37	−36
	Position 3	44.44	54.50	22.92	22.73	9.61	9.41	−6.68	−7.81	−37	−36
